# Supplementary material for: WordCommentsAnalyzer: A windows software tool for qualitative research
Source: F1000Res. 2018 Sep 4;7:536. Originally published 2018 May 3. [Version 2] doi: 10.12688/f1000research.14819.2 (PMC6137411; doi:10.12688/f1000research.14819.2)
Supplement: Supplementary file 3 [file f1000research-7-17614-s0002.tgz › b2a79496-4ada-47c4-9682-a99ce867836d.zip!/Eight Iranian Health Journals/Yafteh _10.docx]

© Yafteh  . This work is licensed under CC BY 4.0 (https://creativecommons.org/licenses/by/4.0/).

Volume & Issue: دوره 17 - شماره 4

Title: ارتباط میان دریافت کلسیم رژیمی و شاخص‌های تن‌سنجی در کارمندان 60-20 ساله دانشگاه علوم پزشکی لرستان در سال 92

http://yafte.lums.ac.ir/article-1-2132-fa.html

مقدمه: شواهد متناقضی در مورد تأثیر دریافت کلسیم بر وزن و درصد چربی بدن وجود دارد. هدف از مطالعه حاضر بررسی ارتباط میان دریافت کلسیم رژیمی و شاخص­های تن­سنجی در افراد بزرگسال بود. مواد و روش­ها: مطالعه مقطعی حاضر روی 94 نفر از کارمندان دانشگاه علوم پزشکی لرستان انجام شد. قد و دور کمر نمونه ­ها به روش استاندارد و وزن، درصد چربی و مساحت چربی احشایی نیز توسط دستگاه  Body Composition Analyzer (ioi 353) اندازه­گیری گردید. جهت تعیین مقدار کلسیم دریافتی، برای هر فرد یک پرسشنامه بسامد خوراک روا و پایا شده تکمیل و اطلاعات وارد برنامهNutritionist 3  شد. برای تجزیه و تحلیل داده­ها از آزمون­های آماری t مستقل، تست فیشر و ضریب همبستگی پیرسون، با استفاده از SPSS19 استفاده شد.  یافته­ ها:  طبق نتایج، 7/44 درصد نمونه­ها دارای وزن طبیعی و 3/55 درصد آن­ها دارای اضافه­وزن و چاقی بودند. میانگین دریافت کلسیم در مردان و زنان به ترتیب 69/982 و 16/1124 میلی­گرم در روز بود. میان کلسیم دریافتی و متغیرهای BMI، دور کمر، درصد چربی بدن و مساحت چربی احشایی در مردان و در زنان، ارتباط معناداری دیده نشد.  بحث و نتیجه­ گیری: بر اساس نتایج، میان دریافت کلسیم رژیمی و شاخص­های تن­سنجی هیچ ارتباط معنی­داری وجود ندارد.

Title: بروز افت تحصیلی و عوامل مرتبط با آن در دانشگاه علوم پزشکی لرستان

http://yafte.lums.ac.ir/article-1-2133-fa.html

مقدمه: افت تحصیلی به معنای عدم موفقیت در تحصیل می باشد که در دانشجویان علوم پزشکی از اهمیت خاصی برخوردار است و بصورت بحرانی تری خود را نشان می دهد. این مطالعه به منظور بررسی میزان افت تحصیلی و عوامل مرتبط با آن در دانشگاه علوم پزشکی لرستان انجام شده است. مواد و روش­ ها: در این مطالعه همگروهی، پرونده تحصیلی کلیه ورودی های 1385 تا 1390 دانشگاه علوم پزشکی لرستان از آموزش کل و معاونت دانشجویی اخذ گردید و اطلاعات جمعیت شناختی و سوابق آموزشی در یک چک لیست وارد گردید. افت تحصیلی بر اساس معدل کل نامطلوب، مشروطی، طولانی شدن مدت تحصیل، اخراج و انصراف از تحصیل تعریف گردید و برای مدل بندی عوامل مرتبط با آن از رگرسیون لوجستیک تحت سطح معناداری 05/0 استفاده شد. یافته­ ها: بروز تجمعی افت تحصیلی در دانشجویان مورد مطالعه 1/25% بود. درضمن عوامل مرتبط با افت تحصیلی شامل دانشکده محل تحصیل، دوره تحصیلی، مقطع تحصیلی، فاصله بین مقاطع تحصیلی قبلی و فعلی، جنسیت و محل سکونت  بوده اند  (05/0>P). بین بروز افت تحصیلی و سهمیه قبولی ارتباط معناداری مشاهده نگردید. بحث و نتیجه­ گیری: مهمترین گروه های در معرض خطر شامل دانشجویان دانشکده پزشکی و بهداشت، دانشجویان دوره شبانه، دانشجویان مقطع کاردانی و دکترای حرفه ای، دانشجویان مذکر، دانشجویان دارای فاصله زیاد بین مقاطع تحصیلی و دانشجویان ساکن خوابگاه دانشجویی بوده اند؛ لذا پیشنهاد می گردد تا  این افراد از ابتدای تحصیل به مراکز مشاوره یا اساتید راهنمای آموزشی معرفی گردند و مورد توجه ویژه قرار گیرند.

Title: بررسی بیوآئروسل های قارچی محیط داخل و فضای آزاد و ارتباط آن با ذرات معلق در یک بیمارستان در شهر خرم آباد

http://yafte.lums.ac.ir/article-1-2136-fa.html

مقدمه : تغییرات آب و هوایی و انتشار ذرات معلق به همراه بیوآئروسل ها به عنوان یک عامل مهم افزایش واکنش های آلرژیک بخصوص در بین بیماران با سیستم ایمنی ضعیف تر معرفی شده است. هدف از این مطالعه بررسی غلظت بیوآئرسل های قارچی در ارتباط با ذرات معلق(pm10,pm1-pm2.5) در بخش های داخلی و فضای آزاد بیمارستان بزرگ آموزشی- درمانی شهر خرم آباد می باشد. مواد و روش ها: در این مطالعه توصیفی-تحلیلی، به مدت 6 ماه نمونه برداری از ذرات معلق و بیوآئروسل های قارچی در 7 بخش داخلی و فضای آزاد بیمارستان مورد بررسی صورت گرفت. در مجموع 192 نمونه (168 نمونه از محیط داخل و 24 نمونه از محیط خارج) برداشت گردید. نمونه برداری از بیوآئروسل های قارچی به روش اندرسون و بوسیله Quick Take 30 با دبی L/min 3/28 و زمان min 5/2 بر روی محیط کشت سابرو دکستروز آگار حاوی کرامفنیکل صورت گرفت. همچنین ذرات معلق نیز با استفاده از Monitor Dust-Trak   مدل 8520 TSI- اندازه گیری شدند. پارامترهای رطوبت نسبی و دما به وسیله دستگاه دیجیتال TES-1360 بررسی شد. یافته ها:  در بین بخش های مورد بررسی، بخش عفونی با CFU/m3 7/101 آلوده ترین بخش بوده و اتاق عمل با CFU/m3 4/46 کمترین آلودگی قارچی را داشت. گونه کلادوسپوریوم با 75/36% بیشترین و گونه رودوتورولا با 7/2% کمترین فراوانی را در مدت نمونه برداری داشتند. نسبت I/O<1  نشان دهنده این است که این آلودگی از محیط خارجی منشاء گرفته است. بحث و نتیجه گیری: بررسی نتایج نشان داد با افزایش رطوبت نسبی و درجه حرارت، تراکم آلاینده های مورد بررسی نیز افزایش می یابد و همچنین بین بیوآئروسل های قارچی و ذرات معلق ارتباط معناداری وجود داشت.

Title: ارتباط بین دریافت رژیمی کلسیم و ویتامین D با سندروم متابولیک و اجزای آن

http://yafte.lums.ac.ir/article-1-2137-fa.html

مقدمه: مطالعات گذشته نشان داده اند که دریافت لبنیات با سندروم متابولیک رابطه معکوسی دارد. گرچه مکانیسم اصلی آن کاملاً روشن نیست، به نظر می رسد کلسیم و ویتامین D دو عامل اصلی مسئول این تأثیرات مثبت باشند. هدف از انجام این مطالعه تعیین ارتباط  بین دریافت کلسیم و ویتامین D رژیمی و سندروم متابولیک و اجزای آن می‌باشد. مواد و روش ها: در این مطالعه مقطعی، 973 نفر( 237 مرد و 736 زن) از ساکنین شهر خرم آباد به روش نمونه گیری خوشه‌ای چند مرحله ای و به صورت تصادفی سیستماتیک انتخاب شدند. دریافت های غذایی افراد با استفاده از پرسشنامه بسامد خوراک (201 آیتمی) ارزیابی و با استفاده از نرم افزار Nutritionist 4 تجزیه و تحلیل شد، نمونه خون سیاهرگی جهت اندازه¬گیری سطح متغیرهای متابولیکی جمع آوری و فشار خون اندازه گیری شد. سندروم متابولیک طبق معیارهای انجمن قلب آمریکا (AHA) تعریف شد. یافته ها: یافته های مطالعه حاضر نشان داد که بین وضعیت تأهل و سندروم متابولیک ارتباط معناداری وجود دارد (001/0=P). همچنین بین تحصیلات و ابتلا به سندروم متابولیک ارتباط معناداری وجود دارد (001/0=P). بین دریافت ویتامین D و افزایش سطح قند خون ناشتا، تری گلیسرید و HDL-کلسترول، ارتباط معکوس معناداری مشاهده گردید (به ترتیب 018/0P=، 04/0 P=، 026/P= ) اما بین کلسیم و اجزای سندروم متابولیک ارتباطی مشاهده نشد. بحث و نتیجه گیری: ارتباط معناداری بین ویتامین D رژیمی و سندروم متابولیک دیده نشد هرچند بین دریافت ویتامین D و خطر بالا بودن سطح قند خون ناشتا، تری گلیسرید و HDL-کلسترول ارتباط معکوسی مشاهده شد. همچنین طبق نتایج به دست آمده بین کلسیم دریافتی و سندروم متابولیک ارتباط معناداری وجود نداشت.

Title: پلی مورفیسم ژن اینترفرون گاما در نقطه T/A 874 در پاسخ به درمان در افراد مبتلا به عفونت هپاتیت C

http://yafte.lums.ac.ir/article-1-2138-fa.html

مقدمه: ویروس هپاتیت C یکی از مشکلات جهانی بهداشت و عامل بیماری مزمن کبدی، سیروز و سرطان کبد می باشد. اینترفرون &alpha; با ریباویرین تنها رژیم درمانی قابل قبول برای این بیماران می باشد که تنها درپنجاه درصد از موارد  موءثرمی باشد. هدف از این مطالعه بررسی پلی مورفیسم ژن اینترفرون گاما در پاسخ به درمان در افراد مبتلا به عفونت هپاتیت C می باشد. مواد و روش ها: این مطالعه بصورت توصیفی - مقطعی بوده که تعداد 78 بیمار تحت درمان در بیمارستان نمازی شیراز در سال90-89، پاسخ یا عدم پاسخ به درمان در آنها مشخص شد. DNA نمونه ها بوسیله نمک استخراج و پلی مورفیسم ژن اینترفرون گاما در منطقه  T/A 874 بوسیله تکنیک  ARMS-PCR  مورد بررسی قرار گرفت. آنالیز آماری داده ها به کمک نرم افزارهای EPI Info2000 و spss16 و با استفاده از آزمون کای دو  مورد تجزیه و تحلیل قرار گرفت. یافته ها:  از بین78 بیمار مورد مطالعه 39 نفر (50 درصد ) دارای آلل TT،  11 نفر (1/14 درصد ) دارای الل AA، 28 نفر(9/39 درصد ) دارای آلل TA بودند. 49 نفر ( 82/62 درصد )  به درمان پاسخ داده و بقیه به درمان جواب نداده ند. فراوانی آلل ها و ژنوتیپ TT بین گروه های مورد مطالعه در عدم پاسخ به درمان معنی دار بود. بحث و نتیجه گیری: اینترفرون گاما یک سایتوکاین مهم در پاسخ سیستم ایمنی در برابر بیماری هپاتیت C می باشد.  پلی مرفیسم در ژن اینترفرون گاما در نقطه ی  T/A 874 می تواند یکی از عوامل مهم ایجاد کننده اختلال در پاسخ به درمان در افراد مبتلا به هپاتیت C باشد.

Title: تأثیر عصاره هیدرو‌الکلی میوه گیاه جغجغه بر میزان گلوکز خون و بیان ژن پیروات کیناز در موش‌های صحرایی دیابتی نوع 1

http://yafte.lums.ac.ir/article-1-2142-fa.html

مقدمه: با توجه به خواص آنتی اکسیدانی عصاره میوه گیاه جغجغه  و نقش آنتی اکسیدان ها در بهبود دیابت، این  مطالعه با هدف اثر عصاره هیدروالکلی میوه گیاه جغجغه بر میزان قند خون و بیان ژن پیروات کیناز در موش های صحرایی دیابتی شده انجام شد. مواد و روش ها: با تزریق استرپتوزوتوسین (mg/kg 60) در  موش های صحرایی نر (300-150 گرم) دیابت نوع 1 ایجاد شد. گروه دیابتی تحت تیمار به صورت روزانه (mg/kg 300) عصاره میوه جغجغه را به صورت  گاواژ  به مدت 30 روز  دریافت نمودند.  گروه شاهد  سالم  و  شاهد  دیابتی  آب مقطر دریافت کردند. سپس در روز قبل از تجویز عصاره  و  روزهای 15  و 30 بعد  از  تجویز  عصاره  میزان گلوکز خون اندازه گیری شد. میزان بیان ژن پیروات کیناز بافت کبدی توسط روش Real-Time PCR بررسی گردید.  یافته ها:  نتایج نشان داد در گروه دیابتی تحت تیمار در روز 15 میزان گلوکز خون به طور معنی داری نسبت به گروه شاهد دیابتی کاهش یافت ولی پس از آن تا پایان دوره مطالعه تغییری در گلوکز خون موش ها مشاهده نشد. بررسی بیان ژن پیروات کیناز نیز نشان داد میزان بیان ژن در گروه دیابتی تحت تیمار در روز 15 به طور معنی داری نسبت به شاهد دیابتی افزایش یافت، سپس تا پایان روز 30 بیان آن تغییر نکرد ولی همچنان از گروه شاهد دیابتی بیشتر بود  بحث و نتیجه گیری: تحقیق حاضر نشان می دهد تجویز عصاره هیدروالکلی میوه گیاه جغجغه توانسته احتمالاً از طریق افزایش بیان ژن پیروات کیناز باعث کاهش گلوکز خون شود.

Title: بررسی ارتباط بین اعتیاد به اینترنت و سلامت عمومی دانشجویان دانشگاه علوم پزشکی لرستان درسال 1392

http://yafte.lums.ac.ir/article-1-2143-fa.html

مقدمه: اﻋﺘﻴﺎد به اﻳﻨﺘﺮﻧﺖ ﺑﻪ ﻣﻌﻨﺎی اﺳﺘﻔﺎده ﺑﻴﺶ از ﺣﺪ ﻳﺎ ﻧﺎ ﻣﻌﻘﻮل و ﺑﻴﻤﺎرﮔﻮﻧﻪ از اﻳﻨﺘﺮﻧﺖ اﺳﺖ که پیامدهای اجتماعی از جمله تاثیر برسلامت عمومی را به دنبال دارد. لذا مطالعه حاضر با هدف بررسی ارتباط بین اعتیاد به  اینترنت و سلامت عمومی دانشجویان دانشگاه علوم پزشکی لرستان درسال 1392 انجام شد. مواد و روش ها: پژوهش حاضر از نوع توصیفی و از نظر نوع بررسی همبستگی می باشد. جامعه آماری آن کلیه دانشجویان )کارشناسی و کارشناسی ارشد و دکترای) دانشگاه علوم پزشکی لرستان می باشد. حجم نمونه بر اساس فرمول و جدول کرجسی مورگان 240 نفر می باشد که به روش نمونه گیری از نوع طبقه ای تصادفی انتخاب شدند. اطلاعات از طریق پرسشنامه شامل پرسش نامه اطلاعات فردی، ﭘﺮﺳﺸﻨﺎﻣﻪ اﻋﺘﻴﺎد ﺑﻪ اﻳﻨﺘﺮﻧﺖ یانگ و پرسشنامه سلامت عمومی گلدبرگ جمع آوری برای تجزیه و تحلیل داده‌ها از آمار توصیفی و استنباطی متناسب و آزمونهای خی 2 – ضریب همبستگی، آزمون های T مستقل، تحلیل واریانس یکطرفه استفاده گردید. آنالیز با استفاده از برنامه آماری SPSS18 انجام گرفت. یافته ها: یافته ها نشان داد که بین نمرات اعتیاد به اینترنت با نمرات سلامت عمومی و مولفه های آن با( ضریب همبستگی471/0- r= در سطح 01/0 P> ) ارتباط معنی داری وجود دارد. از نظر اعتیاد به اینترنت 85/0 درصد دانشجویان نرمال و 15/0 درصد در معرض اعتیاد بودند.  بحث و نتیجه گیری: با توجه به تأثیر اعتیاد به اینترنت بر ابعاد سلامتی دانشجویان، نظارت و کنترل بر استفاده از اینترنت و آگاه نمودن دانشجویان از اثرات مخرب ضروری به نظر می رسد.

Title: شبیه سازی پخش آلاینده های خروجی از دودکش کارخانه سیمان دورود با استفاده از مدل نرم افزاری SCREEN3

http://yafte.lums.ac.ir/article-1-2144-fa.html

مقدمه: با توجه به کاربرد سوخت مازوت در فصل زمستان به عنوان سوخت کوره در کارخانه سیمان دورود و شرایط پایداری هوا و اینورژن دمایی در این فصل، شاهد افزایش غلظت آلاینده ها در اتمسفر این شهر هستیم. بنابراین نیاز به تعیین غلظت این آلاینده ها احساس می گردد. هدف از این پژوهش بررسی پراکنش آلاینده هایNOX ،  SO2و ذرات معلق منتشر شده از دودکش کارخانه  سیمان دورود در پایین دست جهت باد و در فصل زمستان، تا فاصله 5000 متری از منبع انتشار و مقایسه با استاندارد هوای پاک کشور و استاندارد ملی کیفیت هوای آزد (NAAQS) می باشد مواد و روش ها: در این مطالعه، نمونه برداری گازها با استفاده از دستگاه Testo مدل XL 350 و اندازه گیری ذرات خروجی از دودکش کارخانه سیمان نیز توسط دستگاه Westech انجام گرفت. از مدل نرم افزاری SCREEN3 برای بررسی کیفیت هوای محیط و نحوه پراکنش آلاینده های NOX،  SO2و PM استفاده گردید. ورودی مدل شامل میزان غلظت و انتشار گازهای آلاینده، فاکتورهای فیزیکی مرتبط با دودکش کارخانه سیمان، ارتفاع، طول و پهنای ساختمان های در مسیر باد، کلاس پایداری و سرعت باد در ارتفاع 10 متری سطح زمین می باشند. یافته ها:  نتایج حاصل از این مطالعه نشان داد که حداکثر غلظت پیش بینی شده گازهایNOX ، SO2 و PM توسط نرم افزار SCREEN3 در شرایط خنثی(D) و سرعت باد 6 متر بر ثانیه در فاصله 1084 متری دودکش کارخانه سیمان برابر با g/m3µ 48/265، g/m3µ 39/168و g/m3µ 56/56 مشاهده گردیده است. همچنین در شرایط پایدار (F) و سرعت باد 4 متر بر ثانیه، در فاصله 2647 متری دودکش حداکثر میزان غلظت این آلاینده ها به ترتیبg/m3µ 71/195،g/m3µ 54/308 وg/m3µ 73/65 پیش بینی گردیده است. بحث و نتیجه گیری: به منظور دستیابی به استاندارد های هوای پاک، نیاز به پایش مستمر و استفاده از یک سیستم ارتقا یافته جهت کنترل آلاینده های خروجی از دودکش کارخانه سیمان می باشد.

Title: بررسی اثر محافظت نورونی و بهبود رفتاری در موش‌های صحرایی مدل پارکینسونی پیوند شده با سلول‌های بنیادی چربی به صورت داخل وریدی

http://yafte.lums.ac.ir/article-1-2145-fa.html

مقدمه: در بیماری پارکینسون کاهش دوپامین در جسم مخطط منجر به کندی حرکات، سفتی عضلانی و لرزش در هنگام استراحت می گردد. سلول های بنیادی چربی (ADSC) به دلیل دسترسی آسان، ظرفیت تمایزی بالا و به همراه نداشتن مشکلات اخلاقی، مزایای بسیاری در سلول درمانی دارند. در این مطالعه اثر پیوند سلول های ADSC بر اختلال حرکتی موش های مدل پارکینسونی مورد بررسی قرار گرفت. مواد و روش ها: مدل پارکینسونی با تزریق یک طرفۀ µg20 نوروتوکسین 6-OHDA در جسم مخطط موش های صحرایی نر نژاد ویستار ایجاد شد (گروه آسیب). گروه های سلول و MEM-&alpha;  موش های آسیب دیده ای بودند که به آنها به ترتیب106×3 سلول به همراه محیط کشت و محیط تزریق شد. میزان بهبود حرکتی حیوانات با روش روتارود و چرخش های ناشی از آپومورفین در هفته های 4 و8 پس از پیوند سلول ارزیابی شد. یافته ها: به تدریج بر تعداد چرخش خلاف جهت آسیب در گروه¬های آسیب و &alpha;-MEM افزوده شد و در گروه تزریق سلول از تعداد چرخش خلاف جهت آسیب به طور معناداری کاسته شد، در هفته 8 بعد از پیوند اختلاف معنی داری در تعداد چرخش های خلاف جهت آسیب بین گروه سلول با گروه آسیب مشاهده شد. مدت زمان ماندن روی میله در حال چرخش روتارود در گروه آسیب کاهش معناداری در مقایسه با گروه کنترل نشان داد و در گروه سلول افزایش معناداری در مقایسه با گروه آسیب نشان داد. بحث و نتیجه گیری: پیوند داخل وریدی سلول های بنیادی چربی به طور قابل توجهی اختلال حرکتی را در موش های مدل پارکینسونی بهبود می بخشد.

Title: تأثیر یک دوره ماساژ درمانی بر سندرم پای بی قرار

http://yafte.lums.ac.ir/article-1-2146-fa.html

مقدمه: سندرم پای بی قرار نوعی اختلال حرکتی است که با تمایل شدید به حرکت دادن پاها هنگام استراحت مشخص می شود. علی رغم این که منابع علمی تأثیرات بسزای ماساژ را بر برخی بیماری ها نشان می دهد، اما تحقیق جامع و مدونی در خصوص اثر بخشی ماساژ بر سندرم پای بی قرار یافت نمی شود، بنابراین هدف تحقیق عبارت است از تاثیر یک دوره ماساژ درمانی بر سندرم پای بی قرار. مواد و روش­ها: روش مطالعه در این پژوهش از نوع نیمه تجربی، با طرح پیش آزمون و پس آزمون و دارای دو گروه آزمودنی می باشد. گروه تجربی، پیش و پس از مداخله ماساژ درمانی مورد اندازه گیری قرار گرفت و از گروه کنترل فقط اندازه گیری های پیش و پس از آزمون بعمل آمد. جامعه آماری این پژوهش 300 نفر از دانشجویان دختر و پسر (مراکز آموزش عالی استان لرستان) با دامنه نسبی 30-18 سال و با میانگین قد 190-155 سانتی متر و میانگین وزن 85-55 کیلوگرم بود. ابزار تحقیق شامل پرسشنامه محقق ساخته بود که حاوی سوالاتی چهار گزینه ای در ارتباط با علائم بیماری سندرم پای بی قرار بود که شدت اختلال را برحسب درجه بندی خفیف، متوسط، شدید و خیلی شدید نشان می دهد. برای تجزیه و تحلیل آماری از آزمون تی مستقل استفاده شد. یافته ­ها:  نتایج پژوهشی نشان داد که یک دوره ماساژ درمانی بر میزان لنگرمورمور پا، لنگرمیزان تیر کشیدن پا، میزان سوزش پا، میزان پرش ناگهانی پا، میزان خواب وکیفیت خواب آزمودنی هالنگر تأثیر معناداری دارد لنگرلنگر(05/0P<). بحث و نتیجه ­گیری: این بررسی نشان داد که اجرای 8 هفته پروتکل ماساژ درمانی بر کاهش علائم سندرم پای بی قرار تأثیر معنی داری  دارد.

Title: جداسازی و شناسایی باکتری های پروبیوتیکی تولید کننده اسید لاکتیک از شیر الاغ

http://yafte.lums.ac.ir/article-1-2147-fa.html

مقدمه: استفاده شیر الاغ به‌عنوان یک منبع تغذیه  جدید، به دلیل خواص تغذیه ای و فقدان پروتئین های آلرژی زا در حال افزایش است. فلور میکروبی شیر الاغ، به‌خصوص باکتری¬های اسید لاکتیک (LAB) آن هنوز به‌طور کامل شناسایی نشده اند. این تحقیق برای اولین بار با هدف بررسی فلور میکروبی شیر الاغ به‌منظور شناسایی باکتری های بومی با خواص پروبیوتیکی انجام گرفت. مواد و روشها: باکتری های اسید لاکتیک شیر الاغ با استفاده از محیط های کشت اختصاصی و به کمک روش های استاندارد میکروبیولوژی شناسایی گردیدند. تعداد 250 کلونی مجزا و تصادفی از 3 نمونه شیر الاغ مربوط به عشایر شهرستان سلسله جداسازی شدند. برای شناسایی گونه ها از آزمون های گرم، کاتالاز، حرکت در محیط جامد، توانایی رشد در 9/6pH و غلظت (w/v)5/6% کلرید سدیم، تخمیر قندها و آزمون هیدرولیز هیپورات سدیم به همراه شناسایی میکروسکوپی استفاده شد. یافته ها:  از 250 کلونی انتخابی، 20 کلونی، باکتری های کاتالاز منفی، گرم مثبت و کوکسی بودند. نتایج این مطالعه نشان داد که شیر الاغ عمدتاً حاوی گونه هایی از دو جنس استرپتوکوکوس و انتروکوکوس است. بیشترین نوع باکتری جداسازی شده به گونه ی انتروکوکوس فکالیس (حدوداً 55%) تعلق داشتند. برای اولین بار در ایران، نتایج این آزمایش توانست گونه‌ی استرپتوکوکوس دیورسی را از شیر الاغ جداسازی و گزارش نماید. بحث و نتیجه گیری: علیرغم فراوانی لیزوزیم در شیر الاغ، وجود باکتری های پروبیوتیک مفید نشان می دهد که این شیر می‌تواند جایگزین مناسبی برای تغذیه کودکان حساس به پروتئین های شیر گاو باشد. تولید  باکتریوسین ها توسط انتروکوکوس فکالیس، می‌تواند از رشد و تکثیر سایر باکتری های بیماری زا جلوگیری کند. شناسایی گونه‌ استرپتوکوکوس دیورسی در شیر الاغ می‌تواند زمینه را برای مطالعات بعدی در خصوص مشخصات این باکتری در کشور فراهم کند.

Title: اثر حاد تمرین هوازی و تمرین مقاومتی بر لپتین سرم و شاخص مقاومت انسولین در مردان غیر فعال

http://yafte.lums.ac.ir/article-1-2148-fa.html

مقدمه: مقاومت لپتین یکی از موثرترین فاکتورهای هایپر انسولینیمیا است و در نهایت فقدان تحمل گلوکز در اضافه وزن مرتبط با بیماری ها می شود. بنابراین هدف از این تحقیق مطالعه اثر یک تمرین هوازی در برابر تمرین مقاومتی بر لپتین سرم و شاخص مقاومت انسولین در مردان غیر فعال است. مواد و روش ها: روش تحقیق از نوع نیمه تجربی بود. 30 مرد غیر فعال بطور تصادفی انتخاب شدند و در سه گروه تمرین هوازی، مقاومتی و کنترل قرار گرفتند. برنامه تمرینی بصورت تمرین هوازی، دویدن روی نوار گردان به مدت30 دقیقه در 60 تا 70 درصد ضربان قلب ذخیره و تمرین مقاومتی،  با شدت 70 درصد یک تکرار بیشینه با 10 تکرار در هر حرکت برای 3 دور انجام شد. لپتین، گلوکز، انسولین و شاخص مقاومت انسولین قبل و 24 ساعت بعد از فعالیت اندازه گیری شد. یافته ها:  یک جلسه فعالیت مقاومتی همانند فعالیت هوازی تأثیر معناداری در کاهش غلظت لپتین سرم قبل و 24 ساعت پس از فعالیت داشت (p<0/05). همچنین سطوح انسولین سرم و شاخص مقاومت به انسولین در هر دو گروه کاهش معناداری داشت (p<0/05). در حالیکه سطوح گلوکز سرم در هر دو گروه بدون تغییر باقی ماند. بحث و نتیجه گیری: نتایج این مطالعه نشان داد که یک جلسه تمرین مقاومتی همانند تمرین هوازی می تواند با تغییر در سطوح لپتین، اثر مثبتی بر شاخص مقاومت به انسولین در مردان غیر فعال داشته باشد.
